# Readiness for Parkinson’s disease genetic testing and counseling in patients and their relatives in urban settings in the Dominican Republic

**DOI:** 10.1038/s41531-023-00569-y

**Published:** 2023-08-29

**Authors:** Margaret Hackl, Lola Cook, Leah Wetherill, Laurence E. Walsh, Paula Delk, Rebeca De León, Janfreisy Carbonell, Rossy Cruz Vicioso, Priscila Delgado Hodges

**Affiliations:** 1 Department of Medical and Molecular Genetics, Indiana University School of Medicine, Indianapolis, IN USA; 2 Department of Obstetrics and Gynecology, Medical College of Wisconsin, Milwaukee, WI USA; 3 Department of Neurology, Section of Child Neurology, Indiana University School of Medicine, Indianapolis, IN USA; 4 Department of Pediatrics, Indiana University School of Medicine, Indianapolis, IN USA; 5 Parkinson’s Foundation, New York, NY USA; 6 Centro Cardio-Neuro-Oftalmológico y Trasplante (CECANOT), Santo Domingo, Dominican Republic

**Keywords:** Research data, Parkinson's disease

## Abstract

Genetic testing for Parkinson’s disease (PD) is increasing globally, and genetic counseling is an important service that provides information and promotes understanding about PD genetics and genetic testing. PD research studies have initiated outreach to underrepresented regions in North America, including regions in Latin America, such as the Dominican Republic (DR); some studies may include return of genetic test results. Thus, understanding what individuals know about PD, genetic testing for PD, and their interest in speaking with a genetic counselor, is crucial when assessing readiness. In this cross-sectional study, a survey was distributed to people with Parkinson’s disease (PwP) and their unaffected biological relatives in the DR. Questions assessed genetics knowledge, attitude toward genetic testing, and interest in genetic testing and counseling. Of 45 participants, 69% scored the maximum on the attitude scale, indicating an overall positive attitude toward genetic testing; 95% indicated interest in genetic testing for PD, and 98% were at least somewhat interested in meeting with a genetic counselor. The mean PD genetics knowledge score was similar to previously published data. Through free text responses, participants expressed a desire to know more about PD treatment and management, prevention, cause, and their personal risk for PD. These results provide further evidence of readiness for genetic testing in this country but also underscore some gaps in knowledge that should be addressed with targeted educational efforts, as part of building genetic testing and counseling capacities.

## Introduction

Parkinson’s disease (PD) is the second most prevalent neurodegenerative condition, affecting 1 in 100 people over the age of 60^1^ and an estimated 6.1 million individuals worldwide in 2016^2^. Current research suggests that up to 10% of people with PD (PwP) have a monogenic hereditary form of the disease^3,4^. Genetic testing can be used to identify pathogenic variants in PwP, which can provide an explanation for their diagnosis and allow unaffected family members to undergo familial variant testing to assess their own risk to develop PD. Another purpose for genetic testing in PwP is determining eligibility for gene-targeted therapy trials and genomic studies. The landscape of drug development for neurodegenerative diseases is promising, which will hopefully lead to an increase in genetic testing and personalized medicine to identify the most effective treatment for PwP^5,6^.

However, there can be many barriers to accessing genetic testing and counseling and its benefits. Some of these include lack of knowledge, interest, and availability of services, as well as cost^7,8^. Multiple studies have reported a significant interest in genetic testing among PwP and their at-risk family members^9–13^ but have also found participants’ genetics knowledge to be low^9,12,13^. Genetic counseling can address these gaps in knowledge and ensure understanding of the benefits, limitations, and risks of genetic testing for PD^11^. Two studies, to date, have surveyed PwP in the United States (U.S.) about their interest in meeting with a genetic counselor^9,11^ and found that 56% and 43% were interested, respectively. However, the results of these studies are not necessarily generalizable to other populations or predictable. Firstly, the majority of participants in these previous studies were White and well-educated, reducing the usefulness of these results to individuals of differing racial, cultural, and socioeconomic backgrounds. Another consideration is that data from research in Huntington disease suggest interest may not translate into actual uptake of genetic testing and counseling^14,15^, although data from a large clinical trial in North America supports a robust interest and participation in PD testing in North America, with high participant satisfaction, when barriers to testing and counseling are reduced^8^. This clinical trial known as the PD GENEration study (PD GENE) launched in 2019 and offers free genetic testing and counseling in a clinical setting to those with PD. Currently, it is rapidly expanding in the U.S. and into Latin America, including the Dominican Republic (DR), surpassing recruitment goals (https://www.parkinson.org/advancing-research/our-research/pdgeneration). Other international studies, such as GP2, could involve return of PD genetic test results to research participants in Latin American countries in the future (https://gp2.org/).

As genetic testing for PD continues to expand in the DR and other Latin American regions, it is crucial to understand what PwP and their at-risk relatives know about PD, genetic testing for PD, and their interest in speaking with a genetic counselor regarding their risk for PD. This assessment is particularly important to gauge perceptions and readiness in regions where access to and experience with genetics services are limited. Genetic counseling is not yet recognized as an independent profession within the DR or in most of Latin America^16^. As of 2016, only one out of 1,853 public health facilities in the DR had a dedicated clinical genetics service and, outside of this facility, genetic counseling was provided by a limited number of physicians with little to no formal training in medical genetics^17^.

The primary aim of this cross-sectional study was to assess readiness for genetic testing and counseling in PwP and their at-risk relatives in the DR, where access to clinical genetic services, though currently limited^17^, is growing due to new research initiatives^8^. The results of this study will help evaluate the demand for PD genetics services, identify gaps in genetics knowledge, and inform educational strategies and capacity-building within the DR.

## Results

### Sample characteristics

The survey was distributed to 200 PwP via WhatsApp text blast and to 20 PwP via paper fliers that indicated that PWP or unaffected biological relatives were eligible to participate. PwP were encouraged to share the study invitation with their unaffected biological relatives. We received 45 total responses, yielding a 20.4% response rate. Of the 45 individuals, 25 (56%) self-reported a confirmed medical diagnosis of PD (PwP) and 20 (44%) had only a family history (≥1 blood relative) of PD (unaffected relatives).

Participant demographics and characteristics are summarized in Table 1. The mean age of participants was 53 years (standard deviation [SD] = 15), with 48% identifying as male (*N* = 21/44). The majority (73%, *N* = 33/45,) identified as either Mulatto or Mestizo, and 84% (*N* = 38/45) lived in a city. Approximately one-third (30%, *N* = 13/44) of participants reported primary school as their highest level of education and 28% (*N* = 12/45) had a university or post-graduate degree. Among PwP, the mean age was 60, and mean age at diagnosis was 56 years (SD = 14; range = 20–79). The majority of PwP reported that PD made them feel at least somewhat less independent (72%, *N* = 18/25) and that PD had decreased their enjoyment of life at least somewhat (71%, *N* = 17/24). Most PwP (76%, *N* = 19/25) did not have a family history of PD.

**Table 1:** Participant demographics and characteristics^a^.

Characteristic	Total responses (*N* = 45)	PwP (*N* = 25)	Unaffected relatives (*N* = 20)
Age, years, mean (SD, range)	53 (15, 21–80)	60 (12, 21–80)	44 (14, 22–73)
Age at diagnosis, years, mean (SD, range) (*N* = 24)	–	56 (14, 20–79)	–
Family history of PD (≥1 affected family member)			
Yes	–	6 (24%)	–
No	–	19 (76%)	–
Sex	*N* = 44	*N* = 25	*N* = 19
Male	21 (48%)	13 (52%)	8 (42%)
Female	23 (52%)	12 (48%)	11 (58%)
Race/ethnicity			
White	4 (9%)	3 (12%)	1 (5%)
Mulatto	13 (29%)	9 (36%)	4 (20%
Mestizo	20 (44%)	11 (44%)	9 (45%)
Black	7 (16%)	2 (8%)	5 (25%)
Other: Moreno	1 (2%)	–	1 (5%)
Education	*N* = 44	*N* = 24	*N* = 20
Primary school	13 (30%)	10 (42%)	3 (15%)
Some secondary school	5 (11%)	3 (13%)	2 (10%)
Secondary school diploma	8 (18%)	4 (17%)	4 (20%)
Some university	6 (14%)	3 (13%)	3 (15%)
University degree	10 (23%)	2 (8%)	8 (40%)
Post-graduate degree	2 (5%)	2 (8%)	–
Home geographics			
City/Capital	38 (84%)	19 (76%)	19 (95%)
Small town	3 (7%)	2 (8%)	1 (5%)
Rural area	4 (9%)	4 (16%)	–

*PD* Parkinson’s disease, *PwP* people with Parkinson’s disease, *SD* standard deviation

^a^Total number of responses (*N*) for each feature are noted in the top row where relevant.

### Self-reported genetics familiarity and knowledge

When participants were asked how much they knew about genetics (*N* = 44), 52% reported knowing “very little”, 41% reported knowing “some”, and 7% reported knowing “a lot”. Most participants (75%, *N* = 33/44) said they had heard of genetic testing before, while 33% (*N* = 15/45) said they had heard of genetic counseling. Unaffected relatives were more likely to report knowing about genetics (*p* = 0.048) and more likely to have heard of genetic testing (*p* = 0.020) compared with PwP.

### General genetics and PD genetics knowledge

**Fig. 1:**
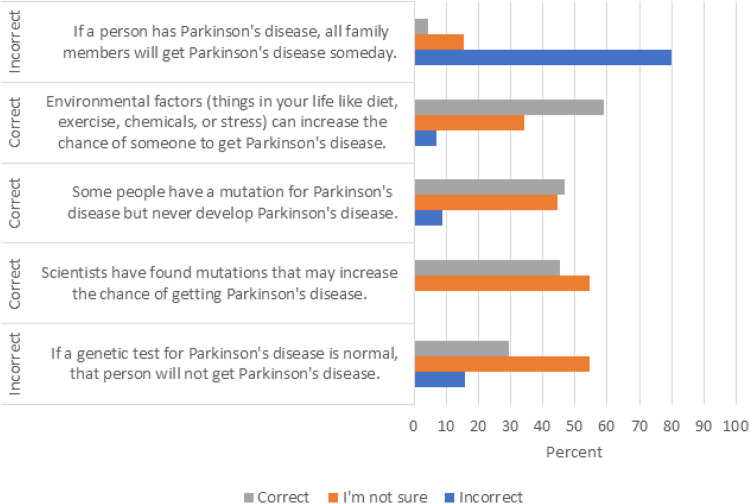
Genetics knowledge of Parkinson’s disease^1^. ^1^*Y*-axis shows if statement was objectively correct or incorrect; *X*-axis shows percentage of participants who responded with each answer choice.

For general genetics knowledge, 76% (*N* = 32/42) of participants scored ≥3 out of a possible 4, putting them in the “high” genetics knowledge category. Most knew that genetic testing may find gene mutations that increase a person’s chance of developing a genetic disease (73%, *N* = 33/45) and that healthy parents could have a child with a genetic disease (77%, *N* = 34/44). Slightly more than half (53%, *N* = 24/45) answered correctly that some people with a genetic mutation may not develop the genetic disease, but 44% (*N* = 20/45) were unsure. The mean PD genetics knowledge score was 2.75 (SD = 1.10) with two participants (5%, *N* = 2/40) receiving the maximum score of 5. More than half (55%, *N* = 24/44) were unsure whether scientists had found mutations that increase the risk of getting PD. About 16% (*N* = 7/44) knew it was incorrect that a normal genetic test for PD meant that person would not get PD. Most participants knew it was incorrect that if a person has PD, all their family members will get PD one day (80%, *N* = 36/45). Participants who self-reported knowing either “some” or “a lot” about genetics had similar PD genetics knowledge scores (mean=3.00, SE = 0.26) as participants who reported knowing “very little” (Wilcoxon *p* = 0.13). There was no association of general genetics knowledge (“high” vs “low” genetics knowledge) or PD genetics knowledge score with any other variables (all *p* > 0.07). PD genetics knowledge results are depicted in Fig. 1.

### Attitude toward genetic testing

**Fig. 2:**
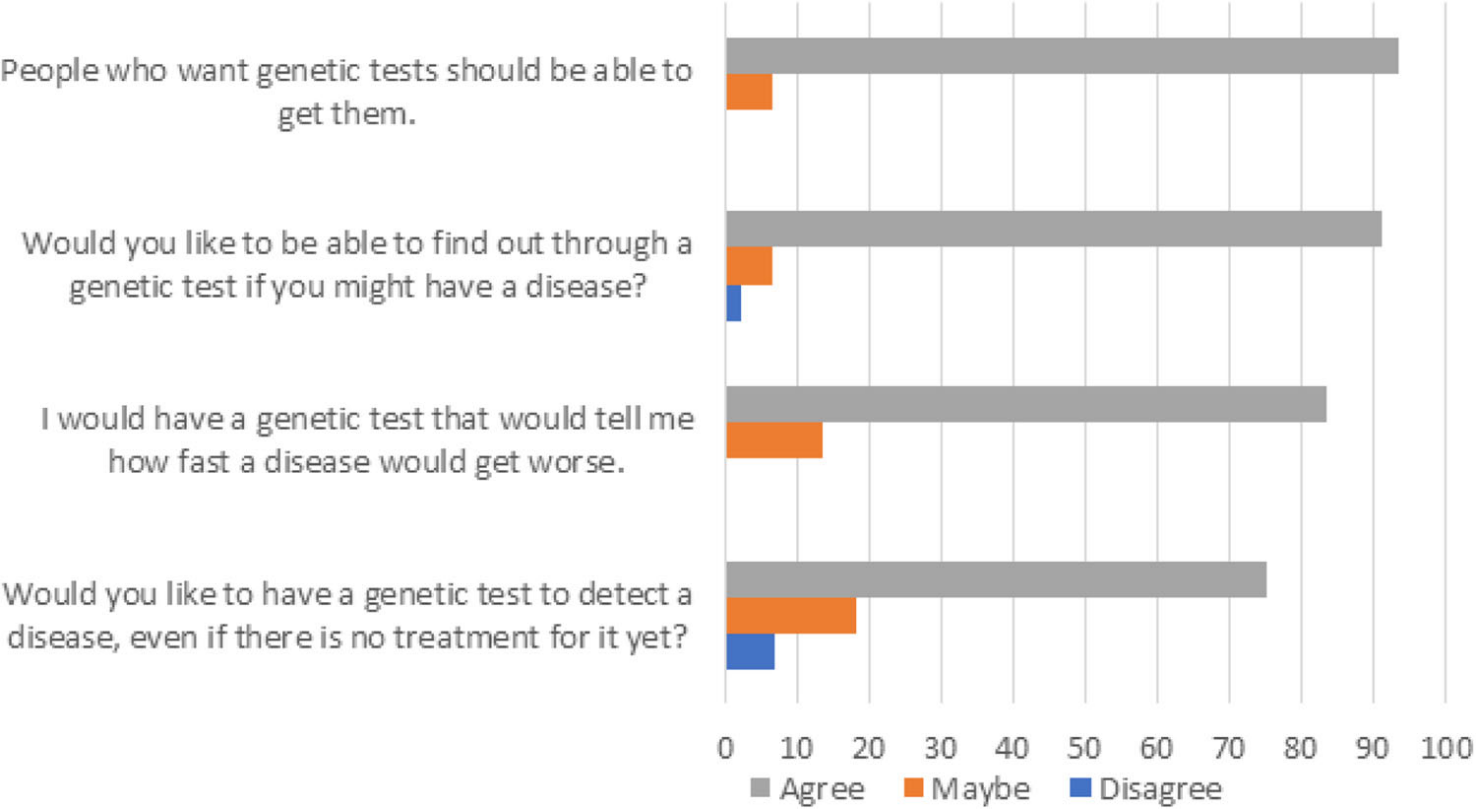
Attitude toward genetic testing^1^. ^1^For each question/statement, Likert scores of 0 for “Disagree”, 1 for “Maybe”, and 2 for “Agree” were summed into a total genetic testing attitude score with a higher score representing a more positive attitude toward genetic testing.

Participants responded to four questions regarding attitude toward genetic testing. Likert responses were summed to represent a total genetic testing attitude score, with a higher score representing a more positive attitude. The mean genetic testing attitude score was 7.3 (SD = 1.34), with 69% of participants scoring the maximum of 8 (*N* = 31/45). Almost everyone (93%, *N* = 42/45) agreed that people who want genetic tests should be able to get them, and that they would like to be able to find out through a genetic test if they might have a disease (91%, *N* = 41/45). The majority (86%, *N* = 38/44) said they would have a genetic test that told them how quickly a disease would progress. One-quarter (25%, *N* = 11/44) of participants were either uncertain or would not want a genetic test for a disease if there was no treatment. Participants who reported primary school as their highest level of education had significantly lower attitude scores than those with a secondary level education or higher (Kruskal–Wallis *p* = 0.031). There was no association of genetic testing attitude score with any other variable (all *p* > 0.07). All results are depicted in Fig. 2.

### Interest in genetic testing and counseling

Seven participants (15%, *N* = 7/45) reported that they had already undergone genetic testing for PD. Almost all participants (95%, *N* = 36/38) without genetic testing expressed at least some interest in doing so, with 76% (*N* = 29/38) being “very interested.” There were three participants (7%, *N* = 3/44) who had already met with a genetic counselor to talk about PD. When the remainder were asked how interested they were in meeting with a genetic counselor, 30% (*N* = 12/40) were “somewhat interested” and 68% (*N* = 27/40) were “very interested.” There was no significant difference between PwP and unaffected relatives in their interest in genetic testing (Fisher’s exact *p* = 0.83) or interest in genetic counseling (Fisher’s exact *p* = 0.30).

### Free text questions

The answers to two free text questions were analyzed via inductive content analysis as described in the “Methods” section. When asked, “What are some ways you think genetic testing for PD would be useful?,” 12 categories were identified among 33 total responses. Responses of “I don’t know” and unintelligible responses were combined into one category that was excluded from further analysis. The remaining 11 categories are presented in Table 2. “Early detection” and “Prevention” were the most common categories with each receiving approximately 21% (*N* = 7/33) of responses. Other common categories included “Develop new treatments,” “Learn about the disease,” and “Guide treatment” (18%, *N* = 6/33 each).

**Table 2:** Summary of participant responses to: *What are some ways you think genetic testing for PD would be useful?*.

Category	Example quotes	*N* (33 total)	%
Early detection	It would be very helpful because they would detect the disease early and treat it at the beginning.	7	21.2
Prevention	If we know in time we have the gene we could make good decisions at an early age to keep exercising or follow medical indications in order to delay the symptoms.	7	21.2
Develop new treatments	This could lead to the development of new formulations and drugs that help to attack the disease more effectively.	6	18.2
Learn about the disease	To detect things about the disease that are not known.	6	18.2
Guide treatment	I believe they help people take the necessary steps to treat the disease.	6	18.2
Determine prognosis	To know how advanced the disease is.	4	12.1
Find the cause	To determine the origin of the disease.	3	9.1
Risk prediction	Through it we can see how big is the possibility of inheriting the disease.	3	9.1
Find a cure	I think it would help to develop medicines to control or ultimately find a cure for this disease.	2	6.1
Help with coping	It could help to better cope with the disease.	1	3
For research	To help in the development of research.	1	3

When asked, “What questions would you want to ask a genetic counselor about PD?,” 17 categories were identified among 38 total responses. Responses of “I don’t know,” “None,” and unintelligible responses were combined into one category that was excluded from further analysis. The remaining 16 categories are presented in Table 3. The category with the highest percentage of responses (18%, *N* = 7/38) was “Treatment/management,” and the next most common category was “Prevention” (16%, *N* = 6/38). All responses to these two questions are available in the Supplementary Results.

**Table 3:** Summary of participant responses to: *What questions would you want to ask a genetic counselor about Parkinson’s disease?*.

Category	Example quote	*N* (total 33)	%
Treatment/management	How to best manage the progression of my disease.	7	18.4
Prevention	How I can avoid it.	6	15.8
Cause of PD	What are the causes of this disease?	5	13.2
Personal risk	How likely are you to develop the disease?	5	13.2
Prognosis	How far do you think your disease might progress and how long would you last with it?	5	13.2
Cure for PD	If with medical indications could it be cured?	5	13.2
Inheritance/Familial risk	Whether children can inherit it.	4	10.5
Support	What can I do to live a normal life?	4	10.5
Implications of positive result	To what degree could a person develop the disease if they had a positive genetic test for Parkinson’s disease for genetic variation?	4	10.5
Detection/diagnosis	If it is possible to have a sure diagnosis.	4	10.5
Symptoms	What are the first symptoms?	3	7.9
PD genetics	To know everything about the genetic part of the disease.	3	7.9
Implications of negative result	To know if I have the test and it is negative if I don’t have the genetic disease.	2	5.3
Updates about PD	I would ask that you keep me up to date on new developments, if possible.	2	5.3
Personal medical question	Why have I lost so much weight even though I eat well and have a good appetite?	1	2.6
Research	Experiments in the search for cures.	1	2.6

*PD* Parkinson’s disease.

### Comparisons of our study responses to Maloney et al. data

The Maloney study^11^ had more males (88% vs 52%, Fisher’s exact *p* = 0.0001), more individuals with at least some college education (89% vs 29%; Fisher’s exact *p* = 0.0001), and more White individuals (86% vs 12%; Fisher’s exact *p* = 0.0001). There was no significant difference in current age (Wilcoxon *p* = 0.54) or age at diagnosis (Wilcoxon *p* = 0.87) between the two study samples. PD genetics knowledge scores were similar between the two samples (Wilcoxon *p* = 0.51). The proportion of PwP in the DR study (74%, *N* = 17/23) who said they were “very interested” in meeting with a genetic counselor was higher than the proportion in the Maloney study who expressed interest in meeting with a genetic counselor (43%) (Fisher’s exact *p* = 0.0006). There was no difference between the two samples regarding genetic testing interest (Fisher’s exact *p* = 0.94).

## Discussion

As PD genetic testing increases in the U.S. and globally, it is important to evaluate readiness in regions where access to clinical genetics services is currently limited but may be expanding. This cross-sectional study aimed to assess general and PD genetics knowledge, attitude toward genetic testing, and interest in genetic testing and counseling among Hispanics in the DR.

Overall, participants expressed an overwhelmingly positive attitude toward genetic testing, in general, and were in favor of open access. Responses to the quantitative attitude assessment indicated strong support for genetic testing in the context of perceived value in personal risk predictions that may accompany genetic testing, as well as determining disease prognoses. A majority of participants remained in support of genetic testing even for conditions where no treatment is available; however, this statement received the largest proportion of “uncertain” or “negative” responses relative to the other attitude statements. While the quantitative attitude assessment was not specific to PD genetic testing, it is important to note that testing does not necessarily predict prognosis and that there are currently no therapeutic treatments available to delay the onset of PD or slow its natural progression^2^. Predictive testing for untreatable neurodegenerative conditions remains controversial and choosing to undergo such testing is a personal decision^18^. Knowledge of genetic status may help inform financial and reproductive planning for those at risk to develop PD, but it may also lead to negative outcomes, including genetic discrimination and psychological distress for individuals and their families^18,19^.

This finding of a generally positive attitude toward genetic testing, but hesitancy toward predictive testing for untreatable conditions, identifies a need for genetic counselors to discuss the benefits, risks, and limitations of genetic testing for PD, especially in asymptomatic individuals. This need is further highlighted by the second free text question to which multiple participants responded with questions related to PD risk assessment and the implications of test results—topics that genetic counselors are trained to handle routinely. Additionally, genetic testing attitude scores were significantly lower for individuals with a primary education compared with those with a secondary education or higher, emphasizing the role that education may play in broadening attitudes toward genetic testing.

Participants expressed an almost unanimous interest in both genetic testing and counseling for PD, despite only about one-third of participants having previously heard about genetic counseling. This illustrates an immense gap between the need for, and interest in, genetic counseling services and local availability in the region^17^. Additionally, we found that the proportion of PwP and family members in our DR cohort that were interested in meeting with a genetic counselor was higher than the proportion of PwP in a primarily White, American cohort^11^. Common PD-related topics that our participants wished to discuss during genetic counseling included:

1. questions about symptoms
2. treatment and management
3. PD causation
4. methods of prevention
5. personal risk assessment
6. inheritance
7. implications of genetic testing results

We also found that individuals in the DR are interested in meeting with a genetic counselor and would likely utilize genetic counseling services if they were readily accessible, supporting the need for additional capacity building for the provision of genetic counseling in the DR. Importantly, participants offered multiple topics of interest that could be personalized for counseling.

Altogether, participants self-reported having generally low genetics knowledge, but unaffected relatives were more likely than PwP to report knowing “a lot” about genetics, which is likely due to differences in age or education level. General genetics knowledge questions assessed for understanding of inheritance, genetic tests, carrier status, and reduced penetrance. Interestingly, the majority of participants demonstrated overall “high” general genetics knowledge by answering at least three out of four of these questions correctly. The most misunderstood genetics concept was reduced penetrance.

Most participants knew that genetic testing could identify mutations that increase a person’s chance of developing a genetic disease, but less than half knew that scientists had found mutations that increase the risk of developing PD. This finding suggests general awareness of inherited diseases, but a lesser realization that PD, specifically, may have a hereditary component. Most participants were familiar with the concept of environmental risk factors for PD but were less certain about the existence of genetic risk factors. Additionally, many individuals were unsure about the significance of a negative genetic test result regarding their personal risk for PD. Free text responses from participants support these findings as multiple individuals expressed a desire to know more about the cause of PD and the genetics of PD. Since PD causation is complex in nature, including multifactorial inheritance and reduced penetrance, this can create difficulties in understanding PD genetics concepts. Although overall knowledge was high, there were misperceptions and gaps as noted above (understanding what causes PD, confusion regarding reduced penetrance, limitations of genetic testing, and the implications of test results including normal results). This could be addressed with educational materials and programs within the DR, targeted to these topics. It is also recommended that providers who counsel PwP and their relatives have awareness of some of these gaps in knowledge, which may extend to individuals outside this region.

It is important to highlight that this study had a small sample size due to time limitations of the project to recruit. Most of our participants lived in a city or urban area, similar to the general population distribution in the DR, where most reside in the cities, and may not accurately represent the perspectives of those living in small towns or rural areas. Nonetheless, it is important to recognize the value of all these perspectives considering the scarcity of research done within this specific population. Another limitation of this study is that a small number of participants had already undergone genetic testing and counseling for PD. These participants likely had a basic education about general and PD genetics, thus, skewing general genetics knowledge and PD genetics knowledge scores for our sample. Self-selecting bias may have also influenced the study results, as participants may have been more interested in PD genetics/counseling, skewing responses to knowledge questions and resulting in greater interest to meet with a genetics specialist. Additionally, the recruitment flier emphasized genetic testing, potentially attracting participants with a higher baseline interest in genetic topics, as evident in some of the free responses. The authors further note that the survey data of affected and unaffected individuals was combined due to the overall sample size and small numbers in each subset. We recognize that this may have blurred some of the unique responses from each group.

The inclusion of unaffected relatives in this study could lead to bias as related family members oftentimes share similar views and experiences. Since responses were anonymous, there was no way to assess how many members of the same family participated in the survey. It was also unfeasible to prevent individuals from taking the survey more than once; however, there were no incentives that might encourage individuals to do so. Additionally, some participants took the survey on an iPad at their in-person neurology visits which could have resulted in response bias. A final limitation of this study is the relatively small number of questions utilized to assess general genetics knowledge and PD genetics knowledge. These sections contained four and five questions, respectively, which may not be enough to assess true understanding of genetics concepts. It is also possible that scores were skewed by guessing, although this was discouraged by including “unsure” as a response option.

In summary, this study revealed an overall positive attitude toward genetic testing and a high interest in both genetic testing and counseling for PD in a DR cohort consisting primarily of Hispanics residing in the city and other urban areas. Interest in these services was either similar to or greater than that of a primarily White study cohort in the U.S., despite no significant differences in PD genetics knowledge between the two. These findings support the need for clinical genetic services in the DR as part of greater capacity building. Researchers may be encouraged to make additional efforts to perform outreach to these underserved populations. An updated study on the current state and availability of genetic services in the DR would be quite valuable, as the most recent review was from 2016^16^.

In addition, our study uncovered questions about PD and key genetics concepts that were commonly misunderstood among PwP and family members in the DR. This may assist providers in delivering tailored genetic counseling to this population and others to ensure understanding of the benefits, limitations, and risks of genetic testing. These results also emphasize a need for genetics education within the DR, targeted to gaps in knowledge. This could be addressed by utilizing Hispanic resources already developed by the author team, including a user-friendly website (https://pdnexus.org) and videos, currently in development, addressing PD genetics misperceptions. Future endeavors based on our findings should include continued expansion of clinical genetics services where resources allow and collaboration with clinicians and researchers in the region to develop and distribute culturally relevant and useful educational materials to both patients and providers to increase knowledge surrounding PD genetics and testing.

## Methods

This study was approved by the Indiana University Institutional Review Board, the Center for Cardio-Neuro-Ophthalmology and Transplantation (CECANOT) Institutional Review Board in Santo Domingo, Dominican Republic, and the Dominican Republic National Council of Bioethics and Health (CONABIOS). Documents required by CECANOT and CONABIOS were submitted in Spanish. This study was conducted with faithful observance to the ethical principles and guidelines stated in the Belmont Report. The authors designed and carried out the study with the utmost consideration of the rights, welfare, and dignity of all participants.

### Participants and recruitment

Recruitment took place via convenience sampling from July 2022 to September 2022. Participants with PD were recruited through a neurology clinic at CECANOT, which is an urban hospital located in Santo Domingo, DR. Eligibility included (1) a confirmed clinical diagnosis of PD or at least one biological relative with PD, (2) fluency in Spanish, (3) age of ≥18 years, and (4) ability to provide informed consent. Confirmation of all eligibility criteria was based on self-report. Capacity to consent was assessed by asking if the participant had a representative who made medical decisions for them. Electronic consent was obtained via an information page at the beginning of the survey which told participants that by clicking “Next Page” they were agreeing to participate in the study.

All recruitment materials were provided in Spanish. Two different methods were used to recruit participants. Approximately 20 paper invitation fliers were handed out to PwP and any accompanying family members at their in-person neurology appointments. These participants had the option of taking the survey on a personal device at home or on an iPad in the clinic. On the first day that fliers were provided, a text invitation was sent via WhatsApp text blast to a recipient list of 200 PwP inviting them and their unaffected biological relatives to participate. The text invitation and physical fliers contained a link and scannable QR code to access the survey. The survey was open for 10 weeks in total.

### Instrumentation

A quantitative and free text survey was utilized to evaluate general genetics knowledge, PD genetics knowledge, attitude toward genetic testing, and interest in genetic testing and counseling. The survey was created by adapting an existing survey developed by Maloney et al.^11^ and a validated genetics knowledge questionnaire developed by Milo Rasouly et al.^20^. The adapted questions were then combined with novel questions developed by the research team. All questions were translated into Spanish by P.D.H., a native Spanish speaker with experience developing Spanish healthcare materials. The Spanish version of the survey was piloted by multiple native Spanish-speaking individuals, and modifications were made to enhance understanding of the questions. The authors decided to use the more familiar term “mutation(s)” rather than “variant(s)” for survey language. Study data were collected and managed using REDCap (Research Electronic Data Capture) electronic data capture tools hosted at Indiana University^21,22^.

The survey contained 29–34 questions that varied depending on the participant’s diagnostic status and prior experience with genetic testing and counseling. Questions included information about demographics, self-perceived genetics knowledge, general and PD genetics knowledge, and attitude toward genetic testing. Interest in genetic testing and counseling was also assessed. Free text questions asked all individuals in what ways they thought genetic testing for PD would be useful, and a second free text question asked all individuals what questions they would want to ask a genetic counselor about PD. Please refer to the Supplementary Methods for the full questionnaire.

### Statistical analyses

To summarize general genetics knowledge, a four-point scale was developed based on the number of questions participants answered correctly. Participants were then classified as having either “high genetics knowledge,” if they scored ≥3, or “low genetics knowledge,” if they scored ≤2, consistent with the classification designated by Milo Rasouly et al.^20^. We used a five-point PD genetics knowledge score to summarize PD genetics knowledge based on the number of correct responses to the five multiple choice questions. This score ranged from 0 (low genetics knowledge) to 5 (high genetics knowledge). Attitude toward genetic testing was summarized based on the Likert responses for 0 = “negative” responses, 1 = “neutral” responses, and 2 = “positive” responses to each of the four multiple choice questions. This score ranged from 0 (negative attitude) to 8 (positive attitude). When comparing interest in genetic testing and counseling to the sample in Maloney et al.^11^, we used only the most affirmative response (Very interested) among PwP in our sample.

We examined whether the following variables differed significantly between PwP and unaffected biological relatives: self-reported genetics knowledge, familiarity with genetic testing and counseling, general genetics knowledge, PD genetics knowledge, attitude toward genetic testing, genetic testing interest, and genetic counseling interest. We explored if age, education, geographic location, and self-reported genetics knowledge were associated with the “high” vs “low” genetics knowledge. Due to the small sample size, non-parametric statistics were employed for all analyses. To assess factors predicting the PD genetics knowledge score and the genetic testing attitude score, we tested age, sex, education, and geographic location (combining small town with rural) using a Kruskal–Wallis test. All associations between categorical variables were evaluated using a Fisher’s exact test.

We compared interest in genetic testing and counseling among PwP in the DR to the sample in Maloney et al.^11^, using only the most affirmative response (Very interested) from the DR sample. Comparisons of PwP between the DR sample and numbers reported from the Maloney sample were performed using either a Fisher’s exact test (categorical variables) or a non-parametric Wilcoxon test (quantitative variables). Variables compared between these two cohorts included current age, age at diagnosis, PD genetics knowledge, sex, education, race/ethnicity, familiarity with genetic counseling, and interest in genetic testing and counseling. Two-sided *p* values are reported for all analyses. All analyses were performed in SAS v9.4. As this is an exploratory study, we did not correct for multiple testing.

Free text data derived from the two free text questions were analyzed via inductive content analysis^23^. The last author (P.D.H.) compiled responses in an Excel spreadsheet and translated them from Spanish to English. The first author (M.H.) reviewed all translated responses, generated initial codes using open coding, and combined related codes into higher level categories. The second author (L.C.) audited the categories and provided suggestions for recategorization, which were then reviewed by M.H. and, when needed, discussed with L.C. until an agreement was reached. A response could be placed in multiple categories. Responses that were unclear or did not answer the original question were placed into a singular category, which was excluded from further analysis. Frequencies were calculated for all remaining categories.

### Reporting summary

Further information on research design is available in the Nature Research Reporting Summary linked to this article.

### Supplementary information

Supplemental Material

Reporting Summary

## Data Availability

The datasets (original Spanish and translated English datasets) are available upon request from the corresponding author. The data are not publicly available due to privacy or ethical restrictions.
