# Supplementary material for: Readiness for Parkinson’s disease genetic testing and counseling in patients and their relatives in urban settings in the Dominican Republic
Source: NPJ Parkinsons Dis. 2023 Aug 29;9:126. doi: 10.1038/s41531-023-00569-y (PMC10465483; doi:10.1038/s41531-023-00569-y)
Supplement: Supplementary file 1 — Supplemental Material [file 41531_2023_569_MOESM1_ESM.pdf]

Supplementary Methods

En esta sección de la encuesta se le harán algunas preguntas sobre sus antecedentes personales y su experiencia con la enfermedad de Parkinson

¿Qué edad tiene usted?

- Introduzca un valor: _____ → Si se introduce 0-17, se le pedirá que salga de la encuesta.

¿Tiene usted un representante que tome decisiones medicas por usted?

- Sí → Lo sentimos, no puede participar en esta encuesta.
- No

¿Tiene usted un diagnóstico médico confirmado de la enfermedad de Parkinson?

- Sí →La lógica de ramificación le llevará a las siguientes 4 preguntas:

  ¿Qué edad tenía cuando le diagnosticaron la enfermedad de Parkinson?

  - Introduzca un valor: _____

  ¿La enfermedad de Parkinson le ha hecho sentir menos independiente?

  - Mucho
  - Un poco
  - No, para nada

  ¿La enfermedad de Parkinson ha disminuido su habilidad de disfrutar la vida?

  - Mucho
  - Un poco
  - No, para nada

  ¿Tiene uno o más familiares de sangre con la enfermedad de Parkinson?

  - Sí
  - No
- No La lógica de ramificación les llevará a la siguiente pregunta:

  ¿Tiene uno o más familiares de sangre con la enfermedad de Parkinson?
  - Sí
  - No Lo sentimos, no puede participar en esta encuesta.

¿Cuál es su sexo?

- Masculino
- Femenino

¿Cuál de las siguientes opciones describe donde usted vive?

- Ciudad/Capital
- Pueblo
- Zona rural

¿Cuál es su nivel de estudios más alto?

- Escuela primaria
- Algunos estudios de secundaria
- Título de Bachiller
- Algo de universidad
- Título universitario
- Título de postgrado

¿Cómo describiría su raza?

- Amerindio
- Asiático o Isleño del Pacífico
- Negro
- Mestizo
- Mulato
- Blanco
- Otro: Por favor, describa: ___________________

Esta sección explora su familiaridad con la genética, las pruebas genéticas y el asesoramiento genético. Por favor, revise las definiciones que aparecen a continuación y responda a las siguientes preguntas:

Definiciones:

- Los **genes** son las instrucciones que determinan nuestro aspecto físico, cómo debe funcionar nuestro cuerpo y cómo nos vamos a desarrollar. La mitad de nuestras instrucciones genéticas vienen de nuestra madre y la otra mitad de nuestro padre.
- **Genética** es el estudio de nuestra información genética (genes) y de cómo ciertos rasgos se heredan de generación en generación.
- Una **prueba genética** examina la sangre o la saliva para detectar cambios o diferencias en nuestras instrucciones genéticas (genes), denominadas mutaciones. Las mutaciones pueden afectar cómo funciona nuestro cuerpo. Una enfermedad causada por una mutación en nuestros genes se denomina una **enfermedad genética**.
- Un **consejero genético** es un profesional de la salud que se reúne con las personas para explicarles cómo la genética puede causar enfermedades y ofrecerles opciones para las pruebas genéticas. Una reunión con un consejero genético se denomina **asesoramiento genético**.

¿Cuánto sabe usted sobre la genética?

- Mucho
- Algo
- Muy poco

¿Ha oído hablar antes de las pruebas genéticas?

- Sí
- No estoy seguro
- No

¿Ha oído hablar antes del asesoramiento genético?

- Sí
- No estoy seguro
- No

Esta sección explora cuánto sabe usted sobre las pruebas genéticas, las mutaciones y las enfermedades genéticas. Por favor, revise las definiciones si es necesario y marque las siguientes frases con "Correcto", "Incorrecto" o "No estoy seguro".

Definiciones:

- Los **genes** son las instrucciones que determinan nuestro aspecto físico, cómo debe funcionar nuestro cuerpo y cómo nos vamos a desarrollar. La mitad de nuestras instrucciones genéticas vienen de nuestra madre y la otra mitad de nuestro padre.
- **Genética** es el estudio de nuestra información genética (genes) y de cómo ciertos rasgos se heredan de generación en generación.
- Una **prueba genética** examina la sangre o la saliva para detectar cambios o diferencias en nuestras instrucciones genéticas (genes), denominadas mutaciones. Las mutaciones pueden afectar cómo funciona nuestro cuerpo. Una enfermedad causada por una mutación en nuestros genes se denomina una **enfermedad genética**.

Las pruebas genéticas pueden encontrar mutaciones genéticas que una persona puede pasar a sus hijos.

- Correcto
- No estoy seguro
- Incorrecto

Los padres sanos pueden tener un hijo con una enfermedad genética.

- Correcto
- No estoy seguro
- Incorrecto

Las pruebas genéticas pueden encontrar mutaciones genéticas que aumentan la posibilidad de que una persona desarrolle una enfermedad genética.

- Correcto
- No estoy seguro

- Incorrecto

Algunas personas con una mutación genética puede que no desarrollen la enfermedad genética.

- Correcto
- No estoy seguro
- Incorrecto

Esta sección explorará cuánto sabe usted sobre las pruebas genéticas para la enfermedad de Parkinson. Por favor, revise las definiciones si es necesario y marque las siguientes frases con "Correcto", "Incorrecto" o "No estoy seguro".

Definiciones:

- Los **genes** son las instrucciones que determinan nuestro aspecto físico, cómo debe funcionar nuestro cuerpo y cómo nos vamos a desarrollar. La mitad de nuestras instrucciones genéticas vienen de nuestra madre y la otra mitad de nuestro padre.
- Una **prueba genética** examina la sangre o la saliva para detectar cambios o diferencias en nuestras instrucciones genéticas (genes), denominadas mutaciones. Las mutaciones pueden afectar cómo funciona nuestro cuerpo. Una enfermedad causada por una mutación en nuestros genes se denomina una **enfermedad genética**.

Los factores del ambiente (aspectos de su vida como la dieta, el ejercicio, químicos o el estrés) aumentan la posibilidad de que alguien desarrolle la enfermedad de Parkinson.

- Correcto
- No estoy seguro
- Incorrecto

Si una prueba genética de la enfermedad de Parkinson es normal, esa persona no desarrollará la enfermedad de Parkinson.

- Correcto
- No estoy seguro
- Incorrecto

Algunas personas tienen una mutación para la enfermedad de Parkinson, pero nunca desarrollan la enfermedad de Parkinson.

- Correcto
- No estoy seguro
- Incorrecto

Los científicos han encontrado mutaciones que pueden aumentar la posibilidad de padecer la enfermedad de Parkinson.

- Correcto
- No estoy seguro

- Incorrecto

Si una persona tiene la enfermedad de Parkinson, todos los miembros de su familia desarrollarán la enfermedad de Parkinson algún día.

- Correcto
- No estoy seguro
- Incorrecto

Esta sección explora su opinión personal sobre las pruebas genéticas para cualquier enfermedad (no sólo la enfermedad de Parkinson). Por favor, revise las definiciones si es necesario y marque las siguientes frases con "De acuerdo", "No estoy de acuerdo" o "Tal vez".

Definiciones:

- Los **genes** son las instrucciones que determinan nuestro aspecto físico, cómo debe funcionar nuestro cuerpo y cómo nos vamos a desarrollar. La mitad de nuestras instrucciones genéticas vienen de nuestra madre y la otra mitad de nuestro padre.
- Una **prueba genética** examina la sangre o la saliva para detectar cambios o diferencias en nuestras instrucciones genéticas (genes), denominadas mutaciones. Las mutaciones pueden afectar cómo funciona nuestro cuerpo. Una enfermedad causada por una mutación en nuestros genes se denomina una **enfermedad genética**.

Yo me haría una prueba genética que me dijera que tan rápido empeoraría una enfermedad.

- De acuerdo
- Tal vez
- No estoy de acuerdo

¿Le gustaría poder enterarse por medio de una prueba genética si es que podría padecer una enfermedad?.

- De acuerdo
- Tal vez
- No estoy de acuerdo

¿Le gustaría hacerse una prueba genética para detectar una enfermedad, aunque aún no haya tratamiento para la misma?.

- De acuerdo
- Tal vez
- No estoy de acuerdo

Las personas que quieren hacerse pruebas genéticas deberían poder hacerlo.

- De acuerdo
- Tal vez
- No estoy de acuerdo

Esta sección explora su opinión e interés en las pruebas genéticas para la enfermedad de Parkinson. Por favor, revise las definiciones si es necesario y responda a las siguientes preguntas.

Definiciones:

- Una **prueba genética** examina la sangre o la saliva para detectar cambios o diferencias en nuestras instrucciones genéticas (genes), denominadas mutaciones. Las mutaciones pueden afectar cómo funciona nuestro cuerpo. Una enfermedad causada por una mutación en nuestros genes se denomina una **enfermedad genética**.

¿Se ha hecho pruebas genéticas para detectar la enfermedad de Parkinson?

- Sí à La lógica de ramificación le llevará a las siguientes 2 preguntas:

  ¿Ha compartido el resultado de la prueba con su familia?

  - Sí
  - No

  ¿Por qué quiso hacerse las pruebas genéticas para la enfermedad de Parkinson?

  - Escriba su respuesta:

- Noà La lógica de ramificación le llevará a las siguientes 3 preguntas:

  ¿Qué tan interesado está en recibir pruebas genéticas para la enfermedad de Parkinson?

  - Muy interesado
  - Algo interesado
  - No estoy interesado

  Si su prueba genética encontrara una mutación para la enfermedad de Parkinson, ¿se lo diría a su familia?

  - Sí
  - Tal vez
  - No

  ¿De qué manera cree que serían útiles las pruebas genéticas para la enfermedad de Parkinson?

  - Escriba su respuesta:

Esta sección explora su opinión e interés en el asesoramiento genético. Por favor, revise las definiciones si es necesario y responda a las siguientes tres preguntas:

Definiciones:

- Un **consejero genético** es un profesional de la salud que se reúne con las personas para explicarles cómo la genética puede causar enfermedades y ofrecerles opciones para las pruebas genéticas. Una reunión con un consejero genético se denomina **asesoramiento genético**.

¿En qué medida cree que el asesoramiento genético sería útil para alguien que está considerando la posibilidad de hacerse una prueba genética para cualquier enfermedad?

- Muy útil
- Algo útil
- No es útil

¿Se ha reunido con un asesor genético para hablar sobre la enfermedad de Parkinson?

- Sí à Fin de la encuesta
- No à La lógica de ramificación llevará a las siguientes 2 preguntas:

    ¿Qué tan interesado está de reunirse con un consejero genético para hablar sobre la enfermedad de Parkinson?

    - Muy interesado
    - Algo interesado
    - No estoy interesado

    ¿Qué preguntas le gustaría hacer a un asesor genético sobre la enfermedad de Parkinson?

    - Escriba su respuesta:

This section of the survey will ask you a few questions about your personal background and your experience with Parkinson's disease.

How old are you?

- Enter a value: _____ → If 0-17 is entered, they will be prompted to exit survey.

Do you have a representative that makes medical decisions for you?

- Yes → I'm sorry, you are not eligible to take this survey.
- No

Do you have a confirmed medical diagnosis of Parkinson's disease?

- Yes à Branching logic will take them to the following 4 sub-questions:

  How old were you when you were diagnosed with Parkinson's disease?
  - Enter a value: _____

  Has Parkinson's disease made you feel less independent?
  - Very much
  - Somewhat
  - Not at all

  Has Parkinson's disease decreased your enjoyment of life?
  - Very much
  - Somewhat
  - Not at all

  Do you have one or more blood relatives with Parkinson's disease?
  - Yes
  - No

- No à Branching logic will take them to the following sub-question:

  Do you have one or more blood relatives with Parkinson's disease?
  - Yes
  - No [illegible] I'm sorry, you are not eligible to take this survey

What is your sex?

- Male
- Female

Which of the following best describes where you live?

- City/Capital
- Small town

- Rural area

What is your highest level of education?

- Primary school
- Some secondary school
- Secondary school diploma
- Some university
- University degree
- Post-graduate degree

How would you describe your race?

- Amerindian
- Asian or Pacific Islander
- Black
- Mestizo
- Mulato
- White
- Other: Please describe: __________________

This section will explore how familiar you are with genetics, genetic testing, and genetic counseling. Please review the definitions below and answer the following questions:

Definitions:

- **Genes** are the instructions that tell our bodies how to look, how to function, and how to develop. We get half of our genetic instructions from our mom and half from our dad.

- **Genetics** is the study of our genetic information (genes) and how certain traits are inherited from generation to generation.

- A **genetic test** examines blood or saliva for changes or differences in our genetic instructions (genes) called **mutations.** Mutations can affect how our bodies function. A disease that is caused by a mutation in our genes is called a **genetic disease.**

- A **genetic counselor** is a healthcare worker who meets with people to explain how genetics can cause disease and provides options for genetic testing. A meeting with a genetic counselor is called **genetic counseling**.

How much do you know about genetics?

- A lot
- Some
- Very little

Have you heard of genetic testing before?
- Yes
- I'm not sure
- No

Have you heard of genetic counseling before?
- Yes
- I'm not sure
- No

This section will explore how much you know about genetic testing, mutations, and genetic disease. Please review the definitions if needed and mark the following sentences with "Correct", "Incorrect", or "I'm not sure".

<u>Definitions</u>:

- **Genes** are the instructions that tell our bodies how to look, how to function, and how to develop. We get half of our genetic instructions from our mom and half from our dad.

- **Genetics** is the study of our genetic information (genes) and how certain traits are inherited from generation to generation.

- A **genetic test** examines blood or saliva for changes or differences in our genetic instructions (genes) called **mutations.** Mutations can affect how our bodies function. A disease that is caused by a mutation in our genes is called a **genetic disease.**

Genetic testing may find genetic mutations that a person can pass on to his/her children.
- Correct
- I'm not sure
- Incorrect

Healthy parents can have a child with a genetic disease.
- Correct
- I'm not sure
- Incorrect

Genetic testing may find genetic mutations that increase a person's chance of developing a genetic disease.
- Correct
- I'm not sure
- Incorrect

Some people with a genetic mutation may not develop the genetic disease.

- Correct
- I’m not sure
- Incorrect

This section will explore how much you know about genetic testing for Parkinson’s disease. Please review the definitions if needed and mark the following sentences with “Correct”, “Incorrect”, or “I’m not sure”.

Definitions:

- **Genes** are the instructions that tell our bodies how to look, how to function, and how to develop. We get half of our genetic instructions from our mom and half from our dad.

  A **genetic test** examines blood or saliva for changes or differences in our genetic instructions (genes) called **mutations.** Mutations can affect how our bodies function. A disease that is caused by a mutation in our genes is called a **genetic disease.**

Environmental factors (things in your life like diet, exercise, chemicals, or stress) increase the chance for someone to get Parkinson’s disease.

- Correct
- I’m not sure
- Incorrect

If a genetic test for Parkinson’s disease is normal, that person will not get Parkinson’s disease.

- Correct
- I’m not sure
- Incorrect

Some people have a mutation for Parkinson’s disease but never develop Parkinson’s disease.

- Correct
- I’m not sure
- Incorrect

Scientists have found mutations that may increase the chance of getting Parkinson’s disease.

- Correct
- I’m not sure
- Incorrect

If a person has Parkinson’s disease, all family members will get Parkinson’s disease someday.

- Correct
- I’m not sure
- Incorrect

This section explores your personal opinion on genetic testing for any disease (not just Parkinson's disease). Please review the definitions if needed and mark the following sentences with "Agree", "Disagree", or "Maybe".

Definitions:

- **Genes** are the instructions that tell our bodies how to look, how to function, and how to develop. We get half of our genetic instructions from our mom and half from our dad.

- A **genetic test** examines blood or saliva for changes or differences in our genetic instructions genes) called **mutations.** Mutations can affect how our bodies function. A disease that is caused by a mutation in our genes is called a **genetic disease.**

I would have a genetic test that would tell me how fast a disease would get worse.

- Agree
- Maybe
- Disagree

Would you like to be able to find out through genetic testing if you might have a disease?

- Agree
- Maybe
- Disagree

Would you like to have a genetic test to detect a disease, even if there is no treatment for it yet?

- Agree
- Maybe
- Disagree

People who want genetic tests should be able to get them.

- Agree
- Maybe
- Disagree

This section will explore your views and interest in genetic testing for Parkinson's disease. Please review the definitions if needed and answer the following questions.

Definitions:

- A **genetic test** examines blood or saliva for changes or differences in our genetic instructions (genes) called **mutations.** Mutations can affect how our bodies function. A disease that is caused by a mutation in our genes is called a **genetic disease.**

Have you had genetic testing for Parkinson's disease?

- Yes à Branching logic will take them to the following 2 sub-questions:

  Did you share your test result with your family?
  - Yes
  - No

  Why did you want genetic testing for Parkinson's disease?
  - Type your response:

- No à Branching logic will take them to the following 3 sub-questions:

  How interested are you in receiving genetic testing for Parkinson's disease?
  - Very interested
  - Somewhat interested
  - Not interested

  If your genetic test found a mutation for Parkinson's disease, would you tell your family?
  - Yes
  - Maybe
  - No

  What are some ways you think genetic testing for Parkinson's disease would be useful?
  - Type your response:

This section will ask questions about your views and interest in genetic counseling. Please review the definitions if needed and answer the following three questions:

Definitions:

A **genetic counselor** is a healthcare worker who meets with people to explain how genetics can cause disease and provides options for genetic testing. A meeting with a genetic counselor is called **genetic counseling**.

How useful do you think genetic counseling would be for someone who is considering genetic testing for any disease?

- Very useful
- Somewhat useful
- Not useful

Have you met with a genetic counselor to talk about Parkinson's disease?

- Yes à End of survey
- No à Branching logic will lead to the following 2 sub-questions:

  How interested are you in meeting with a genetic counselor to talk about Parkinson's disease?

    - Very interested
    - Somewhat interested
    - Not interested

  What questions would you want to ask a genetic counselor about Parkinson's disease?

    - Type your response:

Supplementary Results

Responses to Free Text Questions

Question 1:

**¿De qué manera cree que serían útiles las pruebas genéticas para la enfermedad de Parkinson?**	**What are some ways you think genetic testing for Parkinson's disease would be useful?**
"Creo que ayudarían a desarrollar medicinas para controlar o al final buscar curacion [sic] a esta enfermedad, mientras más temprano se detecta mejor el resultado"	*"I think it would help to develop medicines to control or ultimately find a cure for this disease, the earlier it is detected the better the outcome"*
"Para detectar cosas de la enfermedad que no se conocen. Saber si lo tengo"	*"To detect things about the disease that are not known. To know if I have it"*
"Para el conocimiento de la enfermedad y ver si con eso se encuentra forma de controlar la enfermedad"	*"For the knowledge of the disease and to see if it is possible to find a way to control the disease"*
"Serían útil porque a través de esa prueba nos podríamos dar cuenta si se puede proceder a una operación , y no solo esto si no que a través de la misma podemos ver qué tan grande es la posibilidad de heredar la enfermedad"	*"It would be useful because through this test we could find out if we can proceed with an operation, and not only this but through it we can see how big is the possibility of inheriting the disease"*
"Serían muy importante ya que podemos saber de dicha enfermedad y así sabremos como tratarla"	*"It would be very important since we could know about the disease and like this we could know how to treat it"*
"Si sabemos a tiempo si tenemos el gen podríamos tomar buenas decisiones a temprana edad para mantenernos ejercitados o seguir indicación médica a fin de retardar los síntomas"	*"If we know in time we have the gene we could make good decisions at an early age to keep exercising or follow medical indications in order to delay the symptoms"*
"Podría ayudar a sobrellevar mejor la enfermedad o a conocerla y así tener una mejor calidad de vida. También podrían ser puente a desarrollar mejores y más efectivos tratamientos"	*"It could help to better cope with the disease or to learn about it and thus have a better quality of life. It could also be a bridge to developing better and more effective treatments"*
"Porque podemos saber el pronóstico de la enfermedad"	*"Because we could know the prognosis of the disease"*
"Se podría desarrollar más tratamiento para la enfermedad Párkinson"	*"Further treatment could be developed for Parkinson's disease"*
"Considero que ayudan a las personas a tomar medidas necesarias para tratar la enfermedad"	*"I believe they help people take the necessary steps to treat the disease"*

"Bueno yo creo que eso podría ayudar a saber la causa y así poder ayudar a detener la enfermedad"	*"Well I think that it might help to find out the cause and thus help to stop the disease"*
"Para tomar medidas y diagnóstico con tiempo"	*"To take measures and diagnosis in time"*
"De forma preventiva"	*"Preventively"*
"En función de que está demostrado que el esfuerzo físico retrasada que avance, sería más cuidadoso y haría cosas para mejorar"	*"Based on the fact that it has been demonstrated that physical effort slows down the progress I would be more careful and do things to improve"*
"Para entender mejor o ver razones por que me dio"	*"To better understand or see reasons why I got it"*
"Sería de mucha ayuda ya que a través de estas pruebas se podría detectar que tan avanzado o no está la enfermedad y así poder darle un tratamiento adecuado de la misma"	*"It would be very helpful because through these tests it would be possible to detect how advanced or not the disease is and thus be able to give adequate treatment of the same"*
"Sería de mucha ayuda porque detectarían la enfermedad con tiempo y la tratarían desde un principio"	*"It would be very helpful because they would detect the disease early and treat it at since the beginning"*
"Así se podría desarrollar nueva fórmulas y medicamentos que ayuden a atacar la enfermedad de manera más efectiva"	*"This could lead to the development of new formulations and drugs that help to attack the disease more effectively"*
"Saber que tan avanzada esta la enfermedad"	*"To know how advanced the disease is"*
"Porque los demás familiares míos estarían pendiente de los síntomas de su desarrollo"	*"Because my other relatives would be watching for the development of symptoms"*
"Para determinar el origen de la enfermedad, y así poder alguna precaución"	*"To determine the origin of the disease, so that some precaution can be taken"*
"Alertaría al paciente y a la familia para conocerla y preparase para los posibles cambios que puede hacer para tener mejor calidad de vida y contrarrestar la misma"	*"It would alert the patient and family to know about it and prepare them for possible changes they can make to improve their quality of life and counteract it"*
"Ayudar al desarrollo de las investigaciones"	*"To help in the development of research"*
"Poder encontrar tratamientos ajustado a la necesidad personal del paciente, considerando otras manifestaciones de salud"	*"To be able to find treatments adjusted to the patient's personal needs, considering other health concerns"*
"Para detectar mutaciones en las personas que tengan como consecuencia la enfermedad"	*"To detect mutations in individuals that result in disease"*
"Obtener mayor información al respecto"	*"To obtain more information on this matter"*
"Para detectar a tiempo la enfermedad y con esto poder tratarla correctamente"	*"In order to detect the disease in time and thus be able to treat it correctly"*

Question 2:

¿Qué preguntas le gustaría hacer a un asesor genético sobre la enfermedad de Parkinson?	What questions would you want to ask a genetic counselor about Parkinson’s disease?
1. "¿Cómo manejar mejor el avance de mi enfermedad? 2. ¿Qué esperaría con el tiempo que pase con mi cuerpo y mi mente? 3. ¿Cómo influye en mis descendientes el que tenga Parkinson ya que aparentemente está en los genes de mi familia? ¿Hasta que generación podría heredarse? 4. Prevención si ya mis hijos tienen en gen 5. Pedía que me mantenga al día de los nuevos avances, si es posible	*1. How to best manage the progression of my disease 2. What I would expect to happen over time with my body and mind 3. How does it influence my descendants if I have Parkinson's since it is apparently in my family's genes. Prevention if my children already have the gene 5. I would ask that you keep me up to date on new developments, if possible.*
Saber si al hacerme la prueba y está negativa si no tengo la enfermedad genética	*To know if I have the test and it is negative if I don't have the genetic disease.*
Que ¿De cuáles cosas podrían venir la enfermedad? y si podría aportarnos alguna solución	*Which things could cause the disease and if it could provide us with a solution?*
¿Yo que soy hija de una dama con Párkinson qué posibilidad tendría yo de padecer de la enfermedad?	*I am the daughter of a lady with Parkinson's disease, what are my chances of suffering from the disease?*
Que si con indicaciones médica podría llegar a tener cura	*If with medical indications could it be cured*
1. ¿Qué opciones tienen mis descendientes si tuvieran el gen? 2. ¿Qué opciones tengo yo ahora? 3. Me gustaría saber si todos los síntomas que tengo son propios del Parkinson 4. Me sentiría apoyado en este proceso que es bastante incierto (aunque no es una pregunta) 5. Le pediría me mantenga al tanto de cualquier novedad científica que pudiera ayudarme a mi o a mi familia si llegaran a tenerlo.	*1. What options do my descendants (relatives) have if they had the gene 2. What options do I have now 3. I would like to know if all the symptoms I have are typical of Parkinson's 4. I would feel supported in this process which is quite uncertain (although it is was not a question) 5. I would ask you to keep me informed of any scientific developments that might help me or my family if they were to have it.*
¿Cuáles son los primeros síntomas y si existe la manera de cura?	*What are the first symptoms and if there is a way to cure it?*
¿Cuál sería el pronóstico de la enfermedad?	*What would be the prognosis of the disease?*
¿Cuáles serían las consecuencias de las mutaciones genéticas?	*What would be the consequences of genetic mutations?*
Si los hijos lo pueden heredar	*Whether children can inherit it*
¿Cuáles son las causas que ocasionan dicha enfermedad?	*What are the causes of this disease?*

¿Por qué he perdido tanto peso y como bien tengo buen apetito?	*Why have I lost so much weight eventhough I eat well and have a good appetite?*
¿Qué tan rápido se avanza?	*How fast it progresses*
¿Qué tanto cree su enfermedad pueda progresar y que tiempo duraría con ella?	*How far do you think your disease might progress and how long would you last with it?*
Genéricamente [sic] sic ¿de dónde viene la enfermedad de mi padre?	*Genetically where my father's illness comes from?*
La forma de como prolongar la vida de alguien que tenga la enfermedad	*The way to prolong the life of someone who has the disease*
Que ¿Cómo me afecta la genética?	*How genetics affects me?*
¿Podemos padecerlo? Todas sus hijas	*Can we suffer from it? All her daughters*
¿Cuál es la causa de esta enfermedad? ¿Si es genético? ¿Cuál es el tratamiento?	*What is the cause of this disease? is it genetic? and what is the treatment?*
Si lo detectan a tiempo con una prueba genética ¿cómo podría ayudar al paciente eso?	*If detected early with a genetic test, how could that help the patient?*
¿Qué puedo hacer para llevar una vida normal?	*What can I do to live a normal life?*
¿Hasta qué grado podría desarrollar una persona enfermedad si tendría un prueba genética de enfermedad de párkinson positiva para variación genética?	*To what degree could a person develop the disease if they had a positive genetic test for Parkinson's disease for genetic variation?*
¿Cómo se puede determinar a temprana edad? ¿Qué medida se deben tomar para que la enfermedad no avance rápido en el cuerpo?	*How can it be diagnosed at an early age? What measures should be taken so that the disease does not advance rapidly in the body?*
¿Cómo puedo retrasar los síntomas para tener mejor calidad de vida? ¿Cómo puedo detectar la enfermedad, sin tener síntomas avanzado? ¿Cómo puedo cuidar mi parte emocional? Entre otras...	*How can I delay the symptoms to have a better quality of life? How can I detect the disease without having advanced symptoms? How can I take care of my emotional side? Among others...*
¿Es posible tener un diagnóstico seguro?	*Is it possible to have a sure diagnosis?*
Si mi estudio genético salió bien, ¿podrían mis hijos desarrollar la enfermedad?	*if my genetic study "comes out good" could my children develop the disease?*
Los tratamientos. Los métodos se detección de la enfermedad. Experimentación en buscar curas. Los procesos depresivos del Parkinsoniano and otras	*Treatments. Methods of detection of the disease. Experiments in the search for cures. The depressive processes in Parkinson's disease. Other*
Saber todo sobre la parte genética de la enfermedad	*To know everything about the genetic part of the disease*
Conocer más sobre cómo tratar la enfermedad, si hay cura o cómo puedo evitarla	*Learn more about how to treat the disease, if there is a cure or how I can avoid it.*

¿Qué probabilidades hay de que desarrolle la enfermedad?	*How likely are you to develop the disease?*
¿Cómo surge, cómo se puede tratar y evitar?	*How it arises, how it can be treated and avoided?*
Tengo una predisposición genética para esta enfermedad	*I have a genetic predisposition for this disease / *If I have a genetic predisposition for this disease*
Que ¿Cuál sería el pronóstico para tener una mutación genética?	*What would be the prognosis when you have a genetic mutation?*
