# Supplementary material for: Readiness for Parkinson’s disease genetic testing and counseling in patients and their relatives in urban settings in the Dominican Republic
Source: NPJ Parkinsons Dis. 2023 Aug 29;9:126. doi: 10.1038/s41531-023-00569-y (PMC10465483; doi:10.1038/s41531-023-00569-y)
Supplement: Supplementary file 2 — Reporting Summary [file 41531_2023_569_MOESM2_ESM.pdf]

nature portfolio

Corresponding author(s): Priscila Hodges

Last updated by author(s): 8/9/23

Reporting Summary

Nature Portfolio wishes to improve the reproducibility of the work that we publish. This form provides structure for consistency and transparency in reporting. For further information on Nature Portfolio policies, see our Editorial Policies and the Editorial Policy Checklist.

Please do not complete any field with "not applicable" or n/a. Refer to the help text for what text to use if an item is not relevant to your study.
For final submission: please carefully check your responses for accuracy; you will not be able to make changes later.

Statistics

For all statistical analyses, confirm that the following items are present in the figure legend, table legend, main text, or Methods section.

n/a	Confirmed	
☐	☒	The exact sample size (*n*) for each experimental group/condition, given as a discrete number and unit of measurement
☐	☒	A statement on whether measurements were taken from distinct samples or whether the same sample was measured repeatedly
☐	☒	The statistical test(s) used AND whether they are one- or two-sided<br>*Only common tests should be described solely by name; describe more complex techniques in the Methods section.*
☐	☒	A description of all covariates tested
☐	☒	A description of any assumptions or corrections, such as tests of normality and adjustment for multiple comparisons
☐	☒	A full description of the statistical parameters including central tendency (e.g. means) or other basic estimates (e.g. regression coefficient) AND variation (e.g. standard deviation) or associated estimates of uncertainty (e.g. confidence intervals)
☒	☐	For null hypothesis testing, the test statistic (e.g. *F*, *t*, *r*) with confidence intervals, effect sizes, degrees of freedom and *P* value noted<br>*Give P values as exact values whenever suitable.*
☒	☐	For Bayesian analysis, information on the choice of priors and Markov chain Monte Carlo settings
☒	☐	For hierarchical and complex designs, identification of the appropriate level for tests and full reporting of outcomes
☒	☐	Estimates of effect sizes (e.g. Cohen's *d*, Pearson's *r*), indicating how they were calculated

Our web collection on statistics for biologists contains articles on many of the points above.

Software and code

Policy information about availability of computer code

Data collection	We included a sentence stating that data were collected and managed using REDCap.
Data analysis	We included a sentence stating that data were analyzed using SAS v9.4.

For manuscripts utilizing custom algorithms or software that are central to the research but not yet described in published literature, software must be made available to editors and reviewers. We strongly encourage code deposition in a community repository (e.g. GitHub). See the Nature Portfolio guidelines for submitting code & software for further information.

Data

Policy information about availability of data

All manuscripts must include a data availability statement. This statement should provide the following information, where applicable:

- Accession codes, unique identifiers, or web links for publicly available datasets
- A description of any restrictions on data availability
- For clinical datasets or third party data, please ensure that the statement adheres to our policy

The datasets (original Spanish and translated English datasets) are available upon request from the corresponding author. The data are not publicly available due to privacy or ethical restrictions.

Research involving human participants, their data, or biological material

Policy information about studies with human participants or human data. See also policy information about sex, gender (identity/presentation), and sexual orientation and race, ethnicity and racism.

Reporting on sex and gender	Specific sex differences were considered as part of the study design only to assess if differences in our primary variables differed by sex. We also used sex to check for differences between our sample and the Maloney data. These datapoints were collected based on self-report. We did not collect data on gender.
Reporting on race, ethnicity, or other socially relevant groupings	Terms used in the survey to collect Race/Ethnicity were: White; Mulatto (Mixed ancestry - White and Black); Mestizo (Mixed ancestry - Spanish and indigenous); Black; Other. These terms were used because they are recognized and accepted in the Dominican Republic to capture the ancestry of individuals in that country. These terms were reviewed by the collaborators in the study that are from the Dominican Republic. These data points were collected via self-report. We did not utilize race/ ethnicity as a factor or confounding due to the small number of individuals were not Mulatto or Mestizo (7 out of 45).
Population characteristics	Eligibility included 1) a confirmed clinical diagnosis of PD or at least one biological relative with PD, 2) fluency in Spanish, 3) age of ≥ 18 years, and 4) ability to provide informed consent.
Recruitment	Participants with PD were recruited through a neurology clinic at CECANOT, an urban hospital located in Santo Domingo. Two different methods were used to recruit participants. Approximately 20 paper invitation fliers were handed out to people with Parkinson's (PwP) and any accompanying family members at their in-person neurology appointments. On the first day that fliers were provided, a text invitation was sent via WhatsApp text blast to a recipient list of 200 PwP inviting them and their unaffected biological relatives to participate. The text invitation and physical fliers contained a link and scannable QR code to access the survey. In addition, self-selecting bias could have played a role as participants in the study could have been more interested in topics related to PD genetics/counseling therefore increasing the desire to meet with a genetic specialist such as a genetic counselor. In addition, the recruitment flier discusses interest in genetic testing and it possible that those participants responding the survey may have had a higher baseline interest in genetic testing and more question around genetic topics as seen in some of the free response. This limitation is stated in the Limitations section.
Ethics oversight	This study was approved by the Indiana University Institutional Review Board, the Center for Cardio-Neuro-Ophthalmology and Transplantation (CECANOT) Institutional Review Board in Santo Domingo, Dominican Republic, and the Dominican Republic National Council of Bioethics and Health (CONABIOS).

Note that full information on the approval of the study protocol must also be provided in the manuscript.

Field-specific reporting

Please select the one below that is the best fit for your research. If you are not sure, read the appropriate sections before making your selection.

☒ Life sciences ☐ Behavioural & social sciences ☐ Ecological, evolutionary & environmental sciences

For a reference copy of the document with all sections, see nature.com/documents/nr-reporting-summary-flat.pdf

Life sciences study design

All studies must disclose on these points even when the disclosure is negative.

Sample size	Recruitment took place via convenience sampling from July 2022 to September 2022. No sample size calculation was performed. We recognize the study had a small sample size, but as stated in the limitations this project had time limitations.
Data exclusions	No data were excluded from quantitative analyses. Free text responses that were unclear or that did not answer the original question were excluded from qualitative analysis.
Replication	There was no attempt to replicate the findings.
Randomization	There were no experimental groups and no covariates were utilized in analyses.
Blinding	There was no blinding performed in this study due to the fact that there was no allocation and no experimental groups.

Reporting for specific materials, systems and methods

We require information from authors about some types of materials, experimental systems and methods used in many studies. Here, indicate whether each material, system or method listed is relevant to your study. If you are not sure if a list item applies to your research, read the appropriate section before selecting a response.

Materials & experimental systems

n/a	Involved in the study
☒	☐ Antibodies
☒	☐ Eukaryotic cell lines
☒	☐ Palaeontology and archaeology
☒	☐ Animals and other organisms
☒	☐ Clinical data
☒	☐ Dual use research of concern
☒	☐ Plants

Methods

n/a	Involved in the study
☒	☐ ChIP-seq
☒	☐ Flow cytometry
☒	☐ MRI-based neuroimaging

This checklist template is licensed under a Creative Commons Attribution 4.0 International License, which permits use, sharing, adaptation, distribution and reproduction in any medium or format, as long as you give appropriate credit to the original author(s) and the source, provide a link to the Creative Commons license, and indicate if changes were made. The images or other third party material in this article are included in the article's Creative Commons license, unless indicated otherwise in a credit line to the material. If material is not included in the article's Creative Commons license and your intended use is not permitted by statutory regulation or exceeds the permitted use, you will need to obtain permission directly from the copyright holder. To view a copy of this license, visit http://creativecommons.org/licenses/by/4.0/
